# The multifaceted interactions between pathogens and host ESCRT machinery

**DOI:** 10.1371/journal.ppat.1011344

**Published:** 2023-05-04

**Authors:** Yolanda Rivera-Cuevas, Vern B. Carruthers

**Affiliations:** Department of Microbiology and Immunology, University of Michigan Medical School, Ann Arbor, Michigan, United States of America; Joan and Sanford I Weill Medical College of Cornell University, UNITED STATES

## Abstract

The Endosomal Sorting Complex Required for Transport (ESCRT) machinery consists of multiple protein complexes that coordinate vesicle budding away from the host cytosol. ESCRTs function in many fundamental cellular processes including the biogenesis of multivesicular bodies and exosomes, membrane repair and restoration, and cell abscission during cytokinesis. Work over the past 2 decades has shown that a diverse cohort of viruses critically rely upon host ESCRT machinery for virus replication and envelopment. More recent studies reported that intracellular bacteria and the intracellular parasite *Toxoplasma gondii* benefit from, antagonize, or exploit host ESCRT machinery to preserve their intracellular niche, gain resources, or egress from infected cells. Here, we review how intracellular pathogens interact with the ESCRT machinery of their hosts, highlighting the variety of strategies they use to bind ESCRT complexes using short linear amino acid motifs like those used by ESCRTs to sequentially assemble on target membranes. Future work exposing new mechanisms of this molecular mimicry will yield novel insight of how pathogens exploit host ESCRT machinery and how ESCRTs facilitate key cellular processes.

## Overview of the host endosomal sorting complex required for transport machinery

Endosomal Sorting Complex Required for Transport (ESCRT) machinery functions in various cellular pathways ranging from vesicular trafficking and cytokinesis to membrane repair (see reviews [1–6]). The ESCRT machinery (also termed ESCRTs) consists of 4 protein complexes (ESCRT-0, -I, -II, and -III), which sequentially associate with a target membrane and the AAA ATPase vacuolar protein sorting-associated proteins 4A and 4B (VPS4A/B) to mediate membrane deformation and vesicle scission away from the cytosol [7]. Their recruitment to various cellular sites is dependent on ESCRT adaptor proteins or protein complexes that initiate the machinery assembly [6]. For example, in its canonical function involving the formation of multivesicular bodies (MVBs), recruitment of ESCRTs is initiated by ESCRT-0 recognition of ubiquitinated cargo targeted for degradation [5]. **Table 1** summarizes the interactions of complexes that mediate the assembly of the ESCRT machinery. However, other ESCRT functions do not require ESCRT-0 for initiation and instead depend on different adaptors for the initiation of assembly. This is the case for the role of ESCRTs in plasma membrane repair, in which the ESCRT adaptor apoptosis linked gene-2 (ALG-2, also known as PDCD6) interacts with ESCRT components for the repair of injured membranes [6]. Another example is the recruitment of the ESCRTs by the centrosomal protein of 55 kDa (CEP55) during cytokinesis [4]. Due to the multiple functions of ESCRTs in cells, many intracellular pathogens have evolved ways to exploit ESCRTs for key aspects of their life cycles including for pathogen replication, assembly, and egress. This review highlights the clever, and in some cases convergent, strategies by which pathogens make use of the host ESCRT machinery to promote their intracellular survival and propagation.

**Table 1:** Overview of the ESCRT machinery assembly.

Complex	Components	Complex assembly [7–10]
**ESCRT-0**	HRS (HGS) STAM1/2	• HRS interacts with ESCRT-I via the P[S/T]AP motif
**ESCRT-I**	TSG101 VPS28 VPS37 MVB12A, MVB12B, UBA1L, UBAP1, or UMAD1	• UEV domain in TSG101 recognizes HRS P[S/T]AP motif • VPS28 C-terminus interacts with the ESCRT-II
**ESCRT-II**	EAP45 EAP30 EAP20	• EAP45 recognizes VPS28 via the GLUE domain • EAP20 C-terminus interacts with the ESCRT-III
**ESCRT-III**	CHMP1A, CHMP1B CHMP2A, CHMP2B CHMP3 CHMP4A-C CHMP5 CHMP6 CHMP7 IST1	• CHMP6 interacts with EAP20 through the helix-1 domain • CHMP C-terminal MIM domains interact with VPS4 MIT domains
**VPS4**	VPS4A VPS4B	• VPS4 MIT domain recognizes MIM domains in CHMPs
**ESCRT accessory proteins**	ALIX	• ALIX can interact with TSG101 via the P[S/T]AP motif • Bro domain is necessary for the interaction with CHMP4 C-terminus

### Viral late domain motifs provided first insights into how viruses exploit host ESCRTs

The first described interaction of a pathogen with the host ESCRT machinery was identified while studying retroviral budding from cells. Two decades ago, it was discovered that the human immunodeficiency virus-1 (HIV-1) requires host ESCRT components for viral budding [11]. Since then, numerous studies have shed light on the mechanisms by which this important human pathogen exploits the ESCRT machinery of its host [11,12]. A significant breakthrough was the identification of a P[T/S]AP motif in the HIV-1 Gag protein, which functions in the recruitment of the ESCRT-I component tumor susceptibility gene 101 (TSG101) [13]. By doing so, HIV-1 Gag essentially mimics the interaction between the TSG101 UEV domain and the PTAP motif in the ESCRT-0 hepatocyte growth factor-regulated tyrosine kinase substrate protein (HRS/ HGS) [13–16]. This proline-rich sequence, known as a late domain motif, is encoded in the HIV-1 Gag p6 domain that functions at the late stages of the infection cycle, hence the name [17]. Although the PTAP late domain motif was first discovered in HIV-1 Gag, studies have shown that other retroviruses and nonrelated viruses like Ebola virus (EBOV) also use the PTAP late domain for the recruitment of ESCRTs for viral budding [18]. Further studies of retroviral budding by the Rous sarcoma virus (RSV) and the equine infectious anemia virus (EIAV) led to the discovery of other late domain motifs including PPXY and YPX_(n)_L, which also mimic motifs present in ESCRT components. These late domain motifs initiate assembly of the machinery through the binding of the E3-ubiquitin ligase NEDD4 and the ESCRT adaptor protein ALIX, respectively [19–25]. Numerous mammalian viruses have proteins that encode late domain motifs that function as adaptors for the host ESCRT machinery. Intriguingly, viruses can encode multiple late domain motifs within the same adaptor protein, providing evidence for the importance of exploiting this host machinery for their pathogenesis. The newly described sequence motifs PLPPV and FPIV have been shown to also provide late domain activity for viral budding of mouse mammary tumor virus (MMTV) and paramyxoviruses, respectively [26–29]. Although the ESCRT proteins recruited through the PLPPV motif remain to be identified, the FPIV motif encoded in Newcastle disease virus matrix protein (M protein) appears to facilitate an interaction with CHMP4B [30]. Nonetheless, it is well established that viral late domain motifs have evolved to mimic the function of proline-rich motifs that facilitate physiological and structural organization of ESCRT components [8]. **Table 2** provides an overview of the mechanisms for microbial strategies for usurping of the host ESCRTs.

**Table 2:** Strategies of microbial pathogens in exploiting the host ESCRT machinery.

Microbe	Recruitment		Important ESCRT components	Host ESCRT recruitment site	ESCRT function
	Protein	Motif			
**Prokaryotic virus**					
***Sulfolobus turreted icosahedral virus 1* (STIV)**	N.I.*	N.I.	CdvB (ESCRT-III like protein)	Cell membrane	Viral budding [31]
			Vps4A		
**Plant viruses**					
***Tomato bushy stunt virus* (TBSV)**	p33	P[S/T]AP-like motif	Vps23	Peroxisomes	Viral replication complex [32–34]
			Bro1p		
			Vps24		
			Snf7		
			Vps4		
***Carnation Italian ringspot virus*** **(CIRV)**	p36	N-terminus	Vps23	Mitochondria	Viral replication complex [35]
***Brome mosaic virus* (BMV)**	1a	N.I.	Snf7	Endoplasmic reticulum	Viral replication complex [36]
**Retroviruses**					
***Human immunodeficiency virus*** **(HIV-1)**	Gag	P[S/T]AP	TSG101	Plasma membrane	Viral budding [11,13–16,22,37,38,39,40,41]
		YPX_(n)_L	ALIX		
			CHMP2		
			CHMP4		
			VPS4		
***Equine infectious anemia virus*** **(EIAV)**	Gag	YPX_(n)_L	ALIX	Plasma membrane	Viral budding [20,39,41]
			CHMP2		
			CHMP4		
			VPS4		
***Rous sarcoma virus* (RSV)**	Gag	PPXY	NEDD4	Plasma membrane	Viral budding [19,42–46]
		YPX_(n)_L	VPS4		
			TSG101		
**Filoviruses**					
***Ebolavirus* (EBOV)**	VP40	PTAP	TSG101	Plasma membrane	Viral budding [18,38,47–49]
		PPXY	NEDD4		
***Marburg virus* (MARV)**	VP40	PPXY	NEDD4	Plasma membrane	Viral budding [50]
		P[S/T]AP	TSG101		
**Paramyxoviruses**					
***Human parainfluenza virus- 5* (HPIV5)**	M protein	FPIV	?	Plasma membrane	Viral budding [27]
			VPS4		
***Mumps virus* (MuV)**	M protein	FPIV	?	Plasma membrane	Viral budding [28]
			VPS4		
			CHMP4B		
***Newcastle disease virus***	M protein	FPIV	CHMP4B	Plasma membrane	Viral budding [29,30]
**Flaviviridae**					
***Yellow fever virus* (YFV)**	NS3	YPTI?	ALIX	Endoplasmic reticulum	Viral particle assembly [51]
***Japanese encephalitis virus* (JEV)**	NS3	N.I.	TSG101	Endoplasmic reticulum	Viral particle assembly [52,53]
			CHMP2		
			CHMP4		
***Dengue virus* (DENV)**	NS3	YXKT?	TSG101	Endoplasmic reticulum	Viral particle assembly [53,54]
			CHMP2		
			CHMP4		
			ALIX		
***Hepatitis C virus* (HCV)**	NS2 NS5A	N.I.	TSG101	Endoplasmic reticulum	Viral particle assembly [55–58]
			ALIX		
			HRS		
			VPS4		
			CHMP1A		
			CHMP4B		
***Classical swine fever virus* (CSFV)**	NS3	N.I.	TSG101	Endoplasmic reticulum	Viral particle assembly [59,60]
	NS4B		CHMP4B		
	NS5A		CHMP7		
	NS5B		VPS4		
***Tick-borne flaviviruses* (TBFV)**	NS3	LYXLA	ALIX	Replication site	Viral replication [61]
	E- proteins		CHMP4A		
**Other RNA viruses**					
***Hepatitis A virus* (HAV)**	VP2	YPX_(n)_L	ALIX	Multivesicular bodies	Viral release [62–67]
	VP1pX	N.I.	VPS4		
			CHMP1A, B		
			CHMP4B		
			CHMP2A		
***Chikungunya virus* (CHIKV)**	E1	N.I.	HRS	Cytoplasm	Viral particle assembly [68]
	E2		NEDD4		
	nsP4		TSG101		
	nsP1				
	nsP3				
**DNA viruses**					
***Herpes simplex virus 1* (HSV-1)**	UL34	RRRR	ALIX	Nucleus	Nuclear export complex
			CHMP4		
	UL36	P[T/S]AP	TSG101	*Trans*-Golgi	Secondary envelopment [69–75]
		YPX_(n)_L?	CHMP1		
			CHMP4		
			VPS4		
***Epstein–Barr virus* (EBV)**	BFRF1	N.I.	Itch-Ubiquitin ligase	Nucleus	Nuclear export complex [76–78]
			ALIX		
			CHMP4		
			VPS4		
***Human cytomegalovirus* (HMCV)**	N.I.	N.I.	CHMP1A	Endosomal compartment	Secondary envelopment [79]
			VPS4A		
***Vaccinia virus* (VACV)**	F13L	YPX_(n)_L	ALIX	Endosomal compartment and/or *Trans*-Golgi	Viral envelopment and release [80,81]
			TSG101		
			VPS4		
			CHMP1A		
			CHMP3		
			CHMP4C		
			CHMP6		
***Hepatitis B virus* (HBC)**	HBc	N.I.	HRS	Cell periphery	Viral replication and release [82–85]
			VPS28		
			VPS37		
			CHMP1A, B		
			CHMP2A, B		
			CHMP3		
			CHMP4A, B		
			VPS4		
**Intracellular bacteria**					
***Anaplasma phagocytophilum***	N.I.	N.I.	HRS	Pathogen-containing vacuole	Proliferation [86]
			ALIX		
			CHMP4A, B, C		
***Brucella abortus***	N.I.	N.I.	N.I.	Pathogen-containing vacuole	Release [87]
**Uropathogenic *Escherichia coli* (UPEC)**	N.I.	N.I.	ALIX	Pathogen-containing vacuole	Release [88]
			TSG101		
***Mycobacterium tuberculosis***	EsxG	N.I.	HRS	Pathogen-containing vacuole	Lysosome-clearance evasion [89,90]
	EsxH		CHMP1A, B		
			CHMP4B		
***Salmonella enterica***	SpoB	N.I.	HRS	Pathogen-containing vacuole	Pathogen-containing vacuole biogenesis [91–93]
			CHMP1		
			CHMP3		
			CHMP4		
			CHMP5		
			CHMP7		
			VPS4		
***Coxiella burnetii***	N.I.	N.I.	TSG101	Pathogen-containing vacuole	Pathogen-containing vacuole integrity [94]
			CHMP4B		
**Intracellular eukaryote**					
***Toxoplasma gondii***	GRA14	YPX_(n)_L	ALIX	Pathogen-containing vacuole (known as the parasitophorous vacuole or PV)	Vesicular uptake or entrapment within the PV [95]
		P[T/S]AP	TSG101		
			VPS28		
			VPS37A, C		
			UMAD1		
			ALG-2		
			CHMP1A		
			CHMP4B		
			VPS4		
	GRA64	N.I.	TSG101	Pathogen-containing vacuole (known as the parasitophorous vacuole or PV)	Vesicular uptake or entrapment within the PV [96,97]
			VPS37A		
			VPS28		
			UMAD1		
			ALG-2		
			CHMP4B		
	RON4	YPX_(n)_L	ALIX	Plasma membrane	Invasion [98]
	RON5	P[T/S]AP	TSG101		

*N.I., not identified.

### ESCRT homologues in prokaryotic cells and their contributions to phage pathogenesis

Fascinatingly, ESCRT-III and VPS4A homologs can be found in archaea, and there is evidence for the exploitation of these factors by an archaeal virus. In the case of prokaryotes, the ESCRT machinery is significantly reduced. The few homologues identified in *Sulfolobus* spp. correspond to the late acting ESCRT components from ESCRT-III and VPS4 [99,100]. These plant ESCRT homologs are exploited by the archaeal *Sulfolobus* turreted icosahedral virus (STIV) for replication [31]. Although a specific role for the ESCRT-III homologs has not been defined, a similar observation of ring-like structures at budding sites has been described for the archaeal *Sulfolobus* spindle-shaped virus-1 (SSV1) [101].

Homologues of ESCRT components have also been identified in the genomes of Asgard archaea [102]. Genomic analysis of these archaea have identified multiple genes that were thought to be unique to eukaryotes [103]. This novel finding is consistent with Asgard archaea and eukaryotes sharing a common ancestry. Among the eukaryotic signature proteins present in the Asgard archaea superphylum, several are associated with vesicular trafficking, including ESCRT-I-, ESCRT-II-, ESCRT-III- and ubiquitin modifier-like proteins [103]. Functional analysis of these proteins showed that the VPS4A homologue can bind ESCRT-III-like proteins and partially complement the loss of VPS4A in *Saccharomyces cerevisiae* [102]. This has led to recognizing the importance of this ancient machinery in evolutionary distinct cells.

### Plant pathogens and their interactions with their host ESCRT

**Fig 1:**
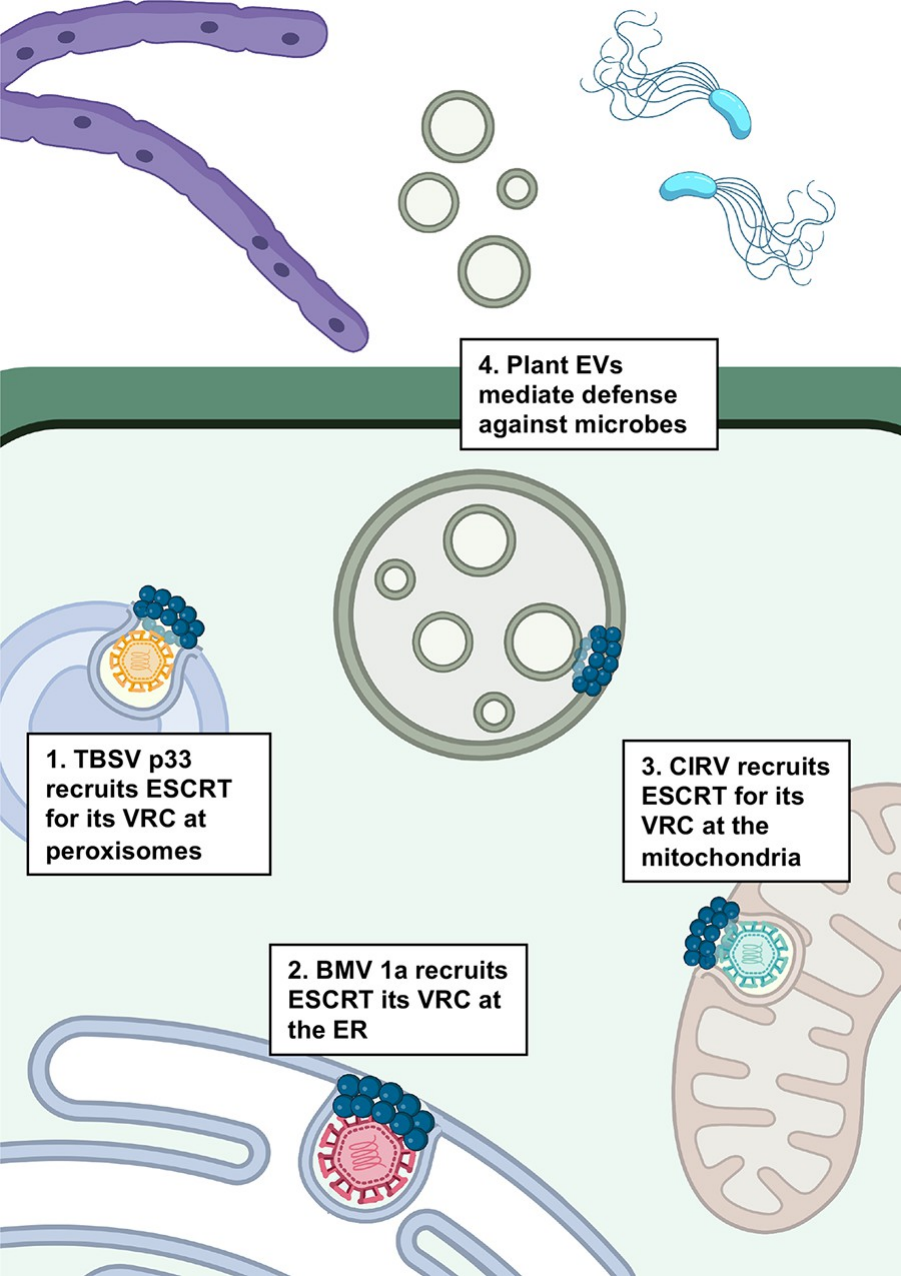
The role of plant ESCRTs in microbial infection. Plant viruses make use of the host ESCRT machinery for the formation of VRCs at different host organelles: (1) TBSV at peroxisomes via the viral protein p33 encoding a P[S/T]AP-like late domain motif; (2) BMV at the mitochondria via the viral protein 1a N-terminus; and (3) CIRV at the ER. (4) Plant ESCRTs also contribute to the formation of EVs that are secreted in response to pathogens as a mechanism for intrinsic defense. Created using Biorender.com. BMV, brome mosaic virus; CIRV, carnation Italian ringspot virus; ER, endoplasmic reticulum; ESCRT, Endosomal Sorting Complex Required for Transport; EV, extracellular vesicle; TBSV, Tomato bushy stunt virus; VRC, viral replication complex.

Although plants lack orthologs of ESCRT-0, they have components of ESCRT-I, -II, and -III that function in endosomal sorting (i.e., MVB biogenesis) and nonendosomal sorting events [104], including exploitation by pathogens (**Fig 1**). The plant virus tomato bushy stunt virus (TBSV) forms viral replication organelles in peroxisomes that are composed of vesicle-like structures termed spherules or viral replication complexes (VRCs), which are coated with the viral replicase protein p33 [105–108]. These structures have been reproduced in yeast to study host factors necessary for their biogenesis [109,110]. A genome-wide screen using this model host identified several ESCRT components that are necessary for TBSV replication [111]. During the formation of VRCs at the peroxisome membrane, p33 is ubiquitinated and recruits the ESCRT-I component Vps23 (TSG101 homologue) through a late domain motif-like sequence, PSVP [112]. Additionally, the ESCRT accessory protein Bro1 (a homologue of human ALIX), the ESCRT-III components Vps24 and Snf7 (homologs of human CHMP3 and CHMP4, respectively), and Vps4 are important for VRC assembly during TBSV infection [32,34]. Surprisingly, host Vps4 appears to remain at the sites of VRCs following recruitment [33], differently from its canonical role in transiently localizing to the site of ESCRT complex assembly to remodel and disassemble the machinery [113]. As a result, the VRC remains “opened” to the host cytosol, and this configuration is also dependent on the function of ESCRT-III components at this structure where the viral RNA is protected from clearance by host intrinsic defenses [33,34].

Other plant viruses that form ESCRT-dependent VRCs at the host mitochondria and endoplasmic reticulum (ER) are the carnation Italian ringspot virus (CIRV) [35] and the brome mosaic virus (BMV) [36,114], respectively. CIRV, a tombusvirus similar to TBSV, interacts with the host ESCRT-I component Vps23 through the viral protein p36 [35]. However, the CIRV p36 protein interacts with Vps23 by “mimicking” the Vps23–Vps28 interaction through the Vps23 StBox (steadiness box) domain [35]. This is different from the TBSV p33 protein and those of other viruses that interact with Vps23 UEV (ubiquitin E2 variant) domain through a P[S/T]AP-like motif [13,18,112]. In the case of BMV, several ESCRT components colocalize with viral protein 1a and are important for the replication of the virus, with host Snf7 (a member of ESCRT-III) depletion having the most significant effect in VRC formation [36].

Another relevant aspect of the ESCRT machinery in plants is its function in forming extracellular vesicles (EVs) and their role in the defense against microbes and facilitating plant–microbe symbiosis, as recently reviewed by Cui and colleagues [115]. Fusion of MVBs with the plasma membrane results in the release of EVs that function in immune defense against plant pathogens [116]. Secreted EVs contain proteins that stimulate fortification of the cell wall as a barrier against pathogens [115]. Plant EVs also contain small RNAs that can target fungal pathogens to induce gene silencing [117]. As a similar mechanism of defense, EV secretion appears to be increased upon *Pseudomonas syringae* infection [118]. However, the role of individual ESCRT components in MVB formation for EV-mediated pathogen defense, and the extent to which this is conserved in mammals, remains to be elucidate.

### Microbial strategies for usurping host ESCRTs in animal cells

#### ESCRT-dependent envelopment: Viral budding at the host plasma membrane

**Fig 2:**
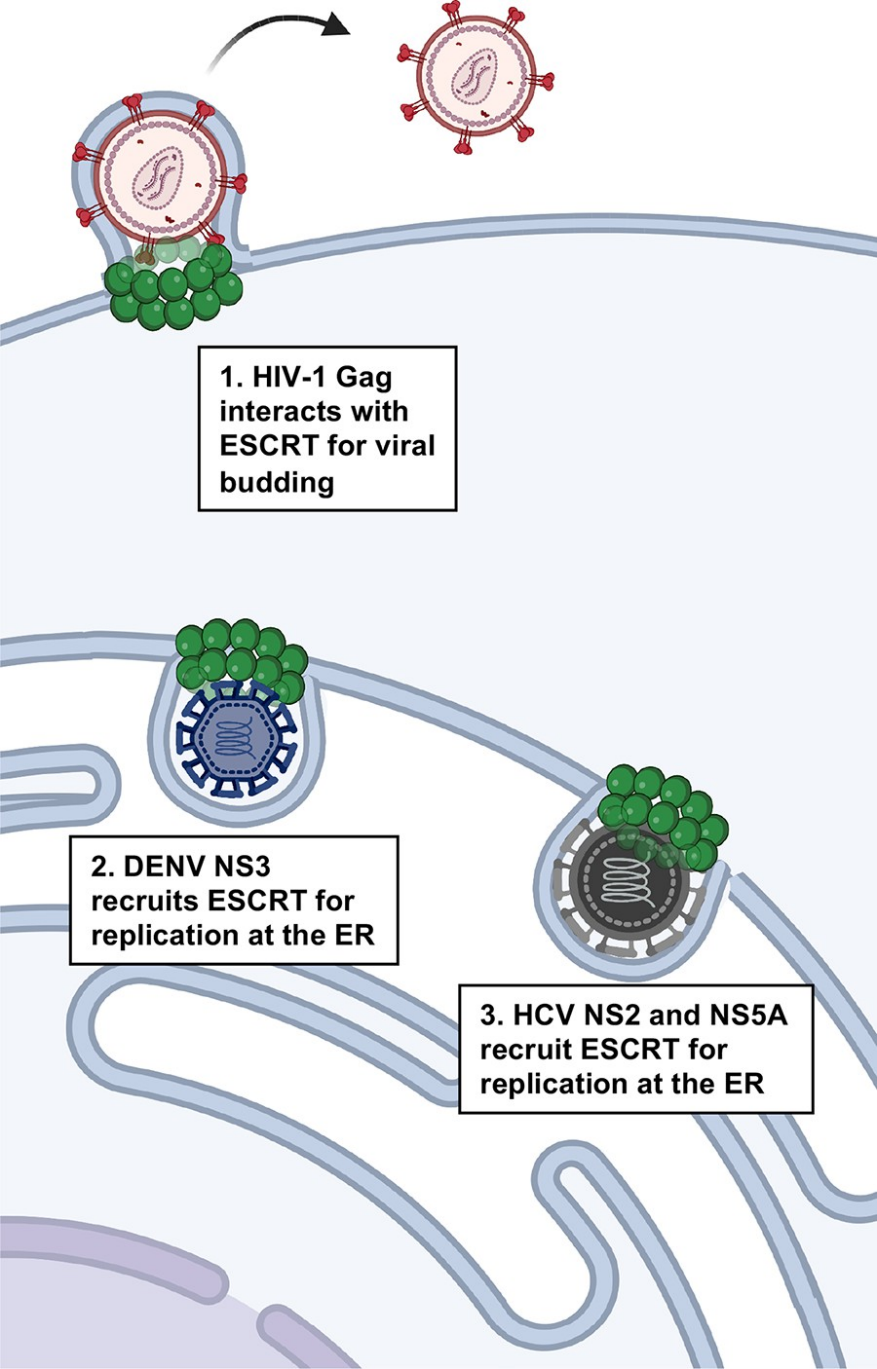
Mechanisms of ESCRT exploitation by RNA viruses. (1) Retroviruses, such as the HIV-1, interact with the host ESCRT machinery for viral budding via structural proteins like Gag, encoding the P[S/T]AP and YPXL late domain motifs. Other RNA viruses like (2) DENV and (3) HCV recruit host ESCRT components to the ER to promote viral replication. This recruitment is facilitated by nonstructural proteins (NS) via unknown mechanisms. Created using Biorender.com. DENV, dengue virus; ER, endoplasmic reticulum; ESCRT, Endosomal Sorting Complex Required for Transport; HCV, hepatitis C virus; HIV-1, human immunodeficiency virus-1.

The budding of enveloped viruses has parallels to ESCRT-dependent MVB formation (**Fig 2**). The role of ESCRTs in this process has been extensively reviewed [119–122]. In some cases, ubiquitination is an important factor for the recruitment of the ESCRTs for viral budding. This is mainly the case for viruses encoding the PPXY late domain motif for the recruitment of the E3-ubiquitin ligase NEDD4, including EBOV, RSV, human T-cell leukemia virus-1 (HTLV-1), murine leukemia virus (MLV), and Marburg virus (MARV) [122]. Ubiquitinated cargo can be recognized by several ESCRT components including the ESCRT-0 HRS protein, the ESCRT-I components TSG101 and Vps23, and the ESCRT accessory protein ALIX [119]. As such, viruses that use the PPXY-NEDD4-ubiquitination strategy have effectively evolved an alternative mechanism for interacting with TSG101 and ALIX in the absence of P[S/T]AP and YPX_(n)_L late domain motifs [123].

Gag is the main structural protein for retrovirus assembly [124]. During HIV-1 release, which is the most studied retroviral ESCRT-mediated budding process, Gag localizes to the plasma membrane of host cells where it interacts with TSG101 through the PTAP motif encoded in its p6 domain [13,15,16,37]. Interestingly, the HIV-1 Gag PTAP peptide binds the host TSG101 UEV domain with a higher affinity than the HRS PTAP peptide [37]. Disrupting either the HIV-1 PTAP motif or the host TSG101 impairs virus release [14,15,17]. Additionally, HIV-1 Gag encodes a second late domain motif, YPX_(n)_L, which binds to the ALIX V domain [39]; however, its binding is weaker than that of the EIAV Gag YPX_(n)_L late domain motif [22]. Nonetheless, ALIX contributes in HIV-1 virus release, and overexpressing it can restore the budding deficiency of HIV-1 PTAP mutants [22,39,40]. ESCRT-III and VPS4 complexes are also critical for HIV-1 viral budding [15,125]. The role of ALIX in viral budding was confirmed using EIAV since this virus lacks a PTAP motif and solely relies on a YPX_(n)_L motif [41].

Recruitment dynamics of ESCRT components to HIV-1 viral assembly sites at the plasma membrane have supported a role for TSG101, ALIX, CHMP4B, and VPS4. Studies using superresolution microscopy provided evidence for the colocalization of TSG101, ALIX, and CHMP4B to the cell membrane where HIV-1 puncta accumulate [126]. These observations were reinforced by live imaging studies [127–129]. Such studies support a model in which HIV-1 Gag recruits TSG101 to the cell plasma membrane where it remains at the site of virus-like particle (VLP) budding, while the late acting ESCRT proteins CHMP4B and VPS4A are transiently recruited to these sites. The recruitment of these late acting ESCRT components is not simultaneous; CHMP4B localized to the nascent VLP a few seconds before VPS4A arrived, but CHMP4B departed within approximately 18 s, whereas VPS4A lingered for approximately 48 s [128,130]. CHMP4B and ALIX assemble simultaneously and their disassembly is dependent on the recruitment and ATPase function of VPS4 [129].

Overall, the viral requirements for early ESCRT components (ESCRT-I, ALIX) appear to vary according to their late domain motifs; however, they converge in the recruitment of CHMP4 and VPS4 isoforms to mediate membrane fission and release of viral particles [41,119].

#### Strategies used by other RNA viruses: Viral particle assembly at intracellular compartments

Although the best described examples of a pathogen usurping the ESCRT machinery are seen in viral egress from the host cell, other RNA viruses have developed their own strategies for manipulating this multifunctional host machinery (**Fig 2**). Different from ESCRT-dependent viral budding, these viruses do not interact with host ESCRTs through their structural proteins. As previously described for plant viruses, animal cell viruses from the *Flaviviridae* family also form cytoplasmic VRCs [131], and their viral nonstructural proteins (NS) are key components for the formation of these important replicative sites [132]. This group of viruses, which includes dengue virus (DENV), Japanese encephalitis virus (JEV), and yellow fever virus (YFV), replicate at VRCs comprised of ER-derived membranous compartments [133].

The role of host ESCRT machinery in *Flaviviridae* pathogenesis remains poorly understood compared to viral budding. However, due to the similarities of their replicative complexes to plant viruses, studies have focused on elucidating the potential role of host ESCRTs in this process. Even though late domain motifs had not been identified in *Flaviviruses* until recently [61], it was known that TSG101 and ALIX can interact with the nonstructural protein NS3 expressed by JEV, DENV, and YFV [51,52,54]. Knocking down TSG101, CHMP2, or CHMP4 reduced JEV and DEV viral titers, and ESCRT-III depletion also affected the number of mature virions particles present at VRCs [53]. Additionally, the protein levels of host ALIX significantly influenced the outcome of viral particles titers in the case of DENV infection [54]. Host CHMP2 and CHMP4 also localized to JEV VRCs, suggesting that they promote membrane deformation for viral particle formation [53].

A more comprehensive depiction of the mechanism for ESCRT-dependent VRC formation was provided in a recent study of classical swine fever virus (CSFV) infection [60]. Using an siRNA screen, the authors identified several ESCRT components that contribute to CSFV infection at different stages of infectious cycle, including entry. Comparable to what has been observed for JEV, the ESCRT-III components CHMP2 and CHMP4 localized to CSFV VRCs [53,60]. Although ESCRT components such as HRS, TSG101, VPS28 (ESCRT-I), EAP20 (ESCRT-II), ALIX, CHMP7, and VPS4 also contribute to VRC formation through their interactions with the nonstructural proteins NS3, NS4B, NS5A, and NS5B [60], the molecular mechanism by which these nonstructural proteins interact with host ESCRT components remains to be elucidated.

Another important human virus within the *Flaviviridae* family is the hepatitis C virus (HCV). Differently from DENV virus, which forms single membrane VRCs, HCV forms ER-derived VRCs composed of double membrane vesicles [134]. Interestingly, HCV also exploits host ESCRTs during viral particle formation [55,58]. In this case, host ESCRTs might be recruited to the site of virus particle assembly through the nonstructural HCV protein NS2 that can interact with the ubiquitin interaction motif domain of the ESCRT-0 component HRS [56]. Since no canonical late domain motifs have been identified in *Flaviviridae* nonstructural proteins discussed here, recruitment of host ESCRTs for VRC formation presumably occurs through novel motifs.

#### DNA viruses: Exploiting the host ESCRT to exit the nucleus

**Fig 3:**
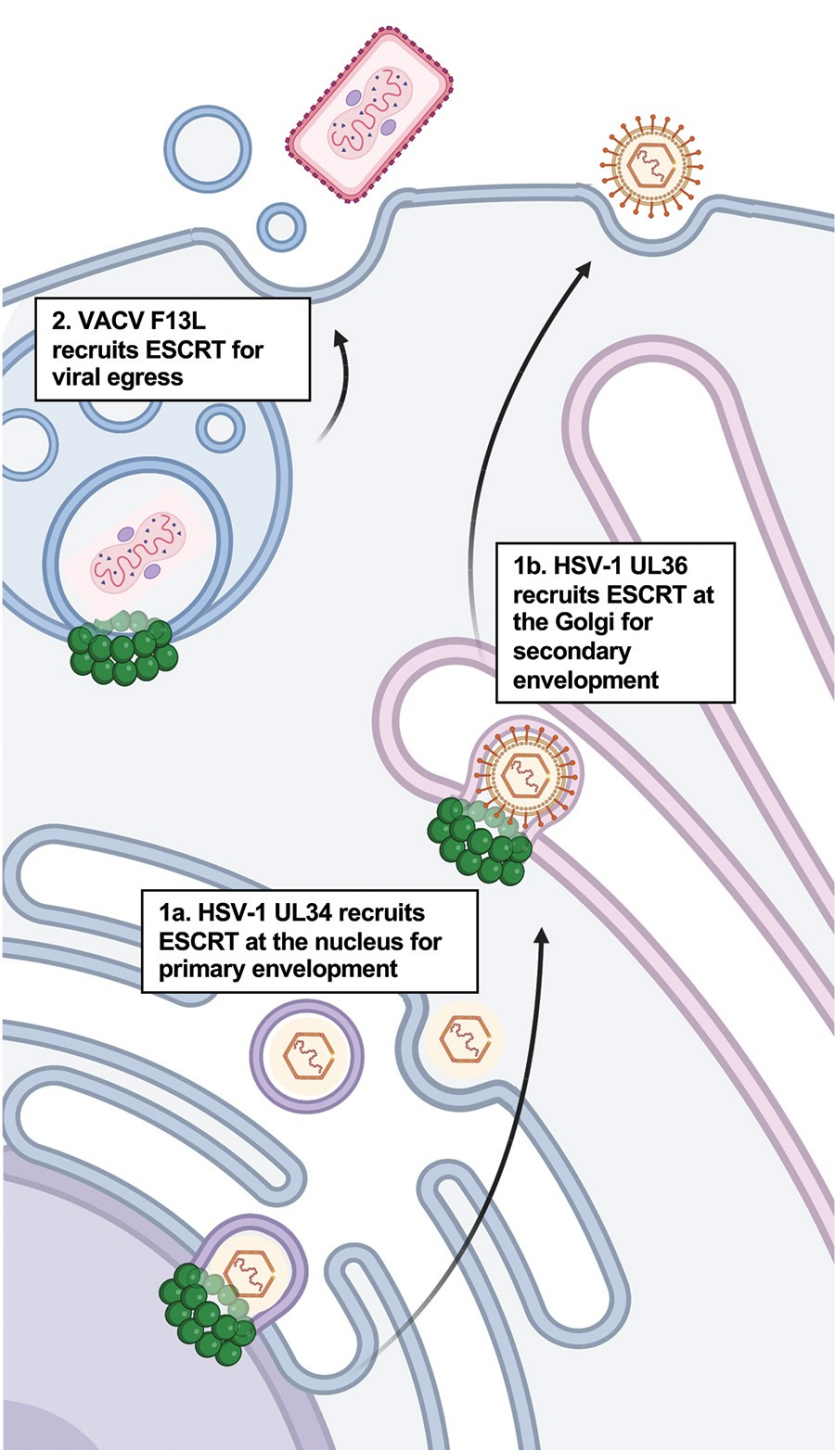
Mechanisms of ESCRT exploitation by DNA viruses. (1) The DNA virus HSV-1 requires ESCRT components for (1a) primary envelopment at the nucleus via the arginine-rich cluster at the UL34 viral protein and (1b) secondary envelopment at the *trans*-Golgi via the P[S/T]AP and YPXL late domain motifs encoded by UL36. (2) VACV recruits the ESCRT machinery for viral envelopment and egress from MVBs. The viral protein F13L has been proposed to interact with ESCRT components via YPXL late domain motifs. Created using Biorender.com. ESCRT, Endosomal Sorting Complex Required for Transport; HSV-1, herpes simplex virus-1; MVB, multivesicular body; VACV, vaccinia virus.

DNA viruses also make use of the host ESCRT machinery at different steps of their infectious cycle (**Fig 3**). In the case of *Herpesviridae* family viruses, there is emerging evidence for a role of host ESCRTs in primary envelopment of herpes simplex virus-1 (HSV-1) and Epstein–Barr virus (EBV) at the inner nuclear membrane (INM) [69,70,76]. There are 2 main proteins in herpesviruses that form the viral nuclear egress complex (NEC), namely UL31 and UL34 in HSV-1, and BFLF2 and BFRF1 in EBV [135]. NECs function in primary envelopment by interacting with the DNA-containing viral nucleocapsid to facilitate budding of enveloped virion from the INM to the perinuclear space [135]. HSV-1 uses UL31 and UL34 to recruit CHMP4 and ALIX to the INM [69]. Knockdown of CHMP4 or ALIX led to the accumulation of virions at the nucleus, providing evidence for a role of the host ESCRT machinery in HSV-1 primary envelopment [69]. Recent work showed that UL34 interacts with ALIX though a novel mechanism involving arginine clusters at its C-terminal [70]. Although these arginine clusters are not conserved in the EBV UL34 homologue BFRF1 [70], a role for BFRF1 in the recruitment of CHMP4 and ALIX has also been reported [76]. BFRF1 recruitment of ESCRT components could be through its ubiquitination, which has been demonstrated to contribute to NEC formation [77]. Although ESCRTs play a basic role in maintaining the nuclear envelope [2], the topology for its proposed role in *Herpesviridae* primary envelopment at the INM (i.e., into the cytosol) is opposite of how it typically functions (i.e., away from the cytosol).

Following HSV-1 capsid release into the host cytoplasm via the fusion of the INM-derived enveloped virion with the outer nuclear membrane (ONM) [135], a second envelopment step occurs at the host *trans*-Golgi [136,137]. Host ESCRT also contributes at this step, as observed by the accumulation of partially enveloped viral particles upon disrupting this machinery using a VPS4 dominant negative (VPS4^EQ^) form [72,73]. The viral protein UL36 (also known as VP1/2), which interacts with TSG101 [74], could be partially responsible for the recruitment of host ESCRTs for secondary envelopment even though depletion of TSG101 does not seem to impair HSV-1 production [73,75]. Remarkably, the HSV-1 viral protein UL51 has structural homology to CHMP4B and is capable of forming ESCRT-III-like filaments [138]. It would be interesting to determine if ESCRT-III components recognize and interact with UL51 filaments to promote viral assembly and the extent to which VPS4 functions in their disassembly.

#### Interactions of intracellular bacteria with host ESCRT

**Fig 4:**
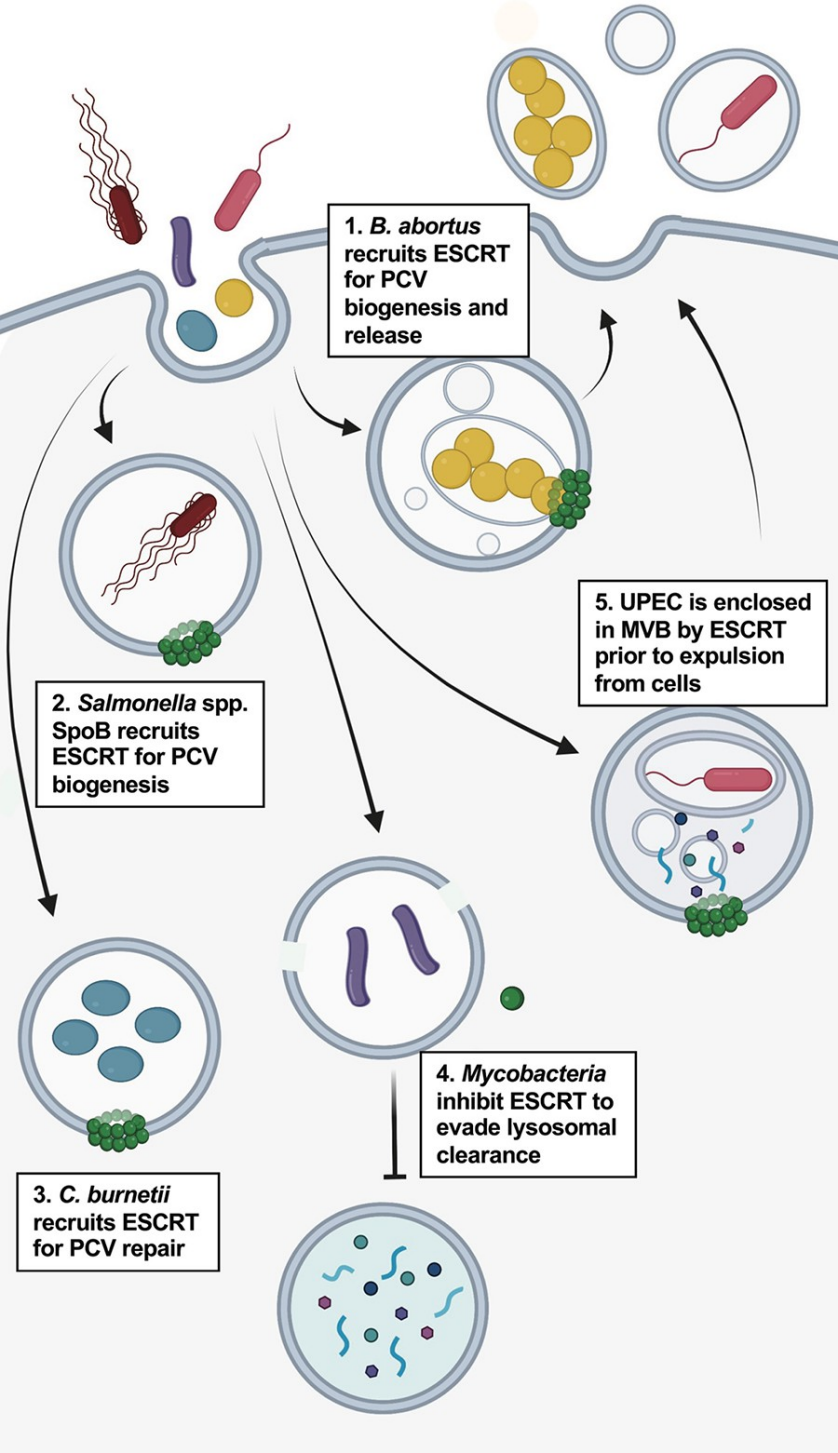
The host ESCRT machinery and nonviral pathogens. The outcome of intracellular bacterial infection is affected by the host ESCRT machinery. (1) *Brucella abortus* recruits ESCRT components to its PVC, which resembles an MVB, to promote proliferation and release. (2) *Salmonella* spp. recruit ESCRT components for the formation of its PCV. The bacterial protein SpoB has been proposed to mediate this interaction. (3) Differently from *A*. *phagocytophilum* and *Salmonella*, *Mycobacterium tuberculosis* inhibits the function of the host ESCRT machinery to evade clearance and promote its survival. The bacteria secrete the effector proteins EsxG and EsxH to inhibit ESCRT-dependent PCV repair (4) The UPEC are encased in MVBs by the host ESCRT machinery for expulsion from cells, a mechanism for ESCRT-mediated intrinsic defense. Created using Biorender.com. ESCRT, Endosomal Sorting Complex Required for Transport; MVB, multivesicular body; PVC, pathogen-containing vacuole; UPEC, uropathogenic *Escherichia coli*.

Most intracellular bacteria reside within a membrane-bound compartment (the pathogen-containing vacuole or PCV) that is modified by the pathogen to promote its survival. Emerging evidence demonstrates that the host ESCRT machinery could also be targeted by intracellular bacteria residing in a PCV **(Fig 4)**. For example, the obligate intracellular bacteria *Anaplasma phagocytophilum* resides within a host cell–derived vacuole that receives membrane traffic from multiple sources, likely to satisfy its metabolic needs [139]. A recent study showed that the *A*. *phagocytophilum* (PCV) resembles an MVB that is decorated with ALIX and ESCRT-0 and ESCRT-III components. Remarkably, knocking down ALIX or components of ESCRT-0 or ESCRT-III not only arrested bacterial growth but also prevented release of progeny by blocking fusion of the PCV with the plasma membrane, which is akin to MVB release of exosomes [86]. This study suggests that *A*. *phagocytophilum* benefits from residing in an MVB-like compartment through the ESCRT-dependent delivery of material and ESCRT-mediated release of infectious progeny. The release of bacteria encased in MVBs has also been previously reported for *Brucella abortus* and uropathogenic *Escherichia coli* (UPEC) [87,88]. It remains to be determined which bacteria effector proteins promote formation of the MVB-like compartment enclosing the bacteria. ESCRT function seems to promote *A*. *phagocytophilum* and *B*. *abortus* progeny release [86,87]; however, in the context of UPEC infection, it has been proposed to be a defense mechanism against the pathogen [88].

Other intracellular bacteria including *Salmonella* spp., *Coxiella burnetii*, and *Mycobacterium tuberculosis* have developed mechanisms to avoid lysosomal dependent clearance and reside in PCVs that promote bacterial persistence and proliferation [140,141]. *Salmonella* can form a PCV in nonphagocytic cells, and a role for host ESCRTs in the biogenesis of its PVC has been recently proposed [91,92]. ESCRT-III components localized to *Salmonella* PCVs, and bacteria invading CHMP3 knockout cells were exposed to the cytosol due to the formation of aberrant PCVs [91]. The ESCRT-III component CHMP4B was also reported to be recruited to the *C*. *burnetii* PCV [94]. In this case, ESCRT recruitment was associated with PCV damage as determined by the co-recruitment of Galectin-3 [94]. CHMP4B and Galectin-3 recruitment to *C*. *burnetii* PCV was transient, potentially corresponding to membrane repair and promoting bacterial survival; this is consistent with the finding that TSG101 depletion reduced bacterial replication [94]. These studies suggest a beneficial role for host ESCRTs in supporting *Salmonella*’s and *C*. *burnetii*’s intracellular niche by contributing to the integrity of the PCV. Conversely, studies of *M*. *tuberculosis* demonstrated that knockdown of ESCRT components increased bacterial growth [89,142], suggesting an antagonistic role for host ESCRT. Additional work showed that host ESCRT machinery is recruited to the mycobacterial PCV in response to membrane damage and that membrane repair promotes lysosomal maturation leading to bacterial clearance [90]. As a mechanism to avoid clearance, mycobacteria effector proteins EsxG and EsxH disrupt host ESCRTs to promote bacterial survival [89,90].

#### An intracellular parasite’s approach for exploiting the host ESCRT machinery

**Fig 5:**
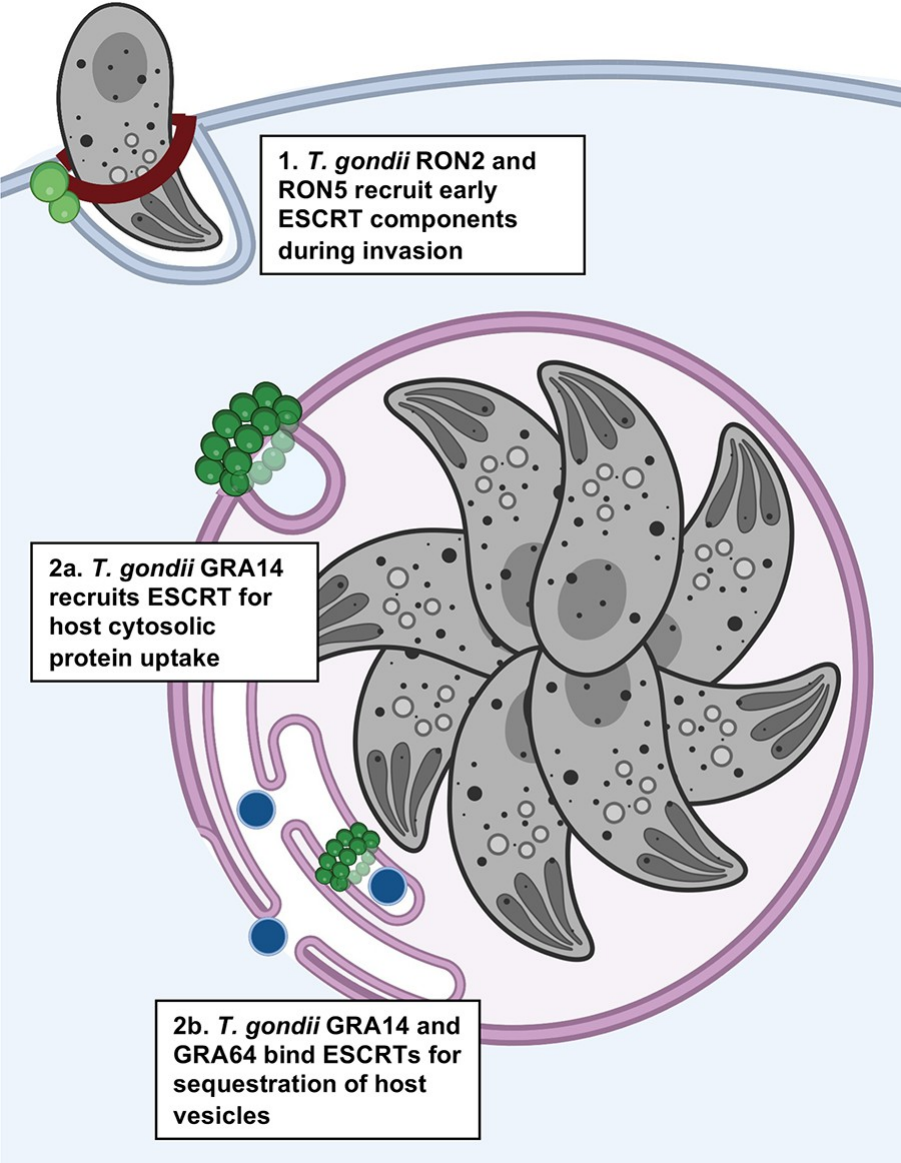
*gondii* has different strategies for exploiting the host ESCRT machinery. ***T*.** (1) The protozoan parasite *Toxoplasma gondii* recruits the early ESCRT components TSG101 and ALIX during invasion via the P[S/T]AP and YPXL late domain motifs encoded by RON5 and RON2, respectively. (2) While residing in its replicative compartment known as the PV, the parasite recruits ESCRT components for the uptake of resources from the host cell across the PV membrane. (2a) GRA14 encodes both P[S/T]AP and YPXL late domain motifs and is required for the recruitment of the host ESCRT machinery for the uptake of host cytosolic proteins. (2b) Additionally, GRA14 alongside another parasite ESCRT-interacting protein GRA64, are necessary for the sequestration of host vesicles. Created using Biorender.com. ESCRT, Endosomal Sorting Complex Required for Transport; PV, parasitophorous vacuole.

Although decades of research described above have identified many different intracellular pathogens that interact with host ESCRTs, a role for ESCRTs during replication of an intracellular eukaryotic microorganism has only recently emerged (**Fig 5**). *Toxoplasma gondii* is an intracellular parasite capable of manipulating the host cell with secretory effector proteins to promote its intracellular survival. One class of effector proteins (GRAs) are secreted from dense granule organelles after the parasite has invaded a cell to create its intracellular niche, the parasitophorous vacuole (PV) [143]. A subset of GRA proteins integrate into the PV membrane with a single transmembrane segment, thereby bridging the PV lumen and host cytosol [144]. Residing at this parasite–host interface, such proteins are ideally positioned to communicate with the infected cell including for the acquisition of resources [145]. *T*. *gondii* acquires proteins and lipids from infected cells by vesicular uptake or entrapment, respectively, within the PV [146,147]. A potential role for the host ESCRT machinery for these processes was hypothesized since it would involve budding of vesicles away from the host cytosol and into the lumen of the PV, consistent with ESCRT function. Accordingly, a recent study identified several ESCRT proteins associated with the PV based on proximity labelling experiments [148]. Additional work showed that ESCRT-III accumulates at the PV upon overexpression of dominant negative VPS4A [95]. Bioinformatic searches identified a PV membrane resident secretory protein called GRA14, which encodes several ESCRT late domain motifs in its C-terminus that are exposed to the host cytosol. Further studies established that GRA14 interacts with the host ESCRT machinery through its late domain motifs to mediate the vesicular uptake of host cytosolic proteins across the PV membrane [95]. Interestingly, recruitment of TSG101, but not ALIX, is dependent on GRA14, suggesting the contribution of other parasite effector proteins in the recruitment of the multiple ESCRT components observed at the PV [95]. The transmembrane dense granule protein GRA64 is a candidate for ALIX recruitment since it was shown to co-immunoprecipitate with ESCRT components [96]. Although GRA64 lacks apparent late domain motifs, it has an arginine cluster like that of the HSV-1 UL34 protein [96]. Moreover, Romano and colleagues [97] recently reported that parasites lacking GRA64 or GRA14 entrap fewer intra-PV host organelles. Disrupting both GRA64 and GRA14 resulted in an additive effect, suggesting that these proteins have nonredundant functions. This study also showed that CHMP4B is recruited to the PV and that CHMP4B forms striking spiral filament within PV membrane invaginations when expressed as a dominant negative mutant. The authors further report that expression of dominant negative VPS4A caused the accumulation of entrapped host-derived endolysosomal vesicles in the PV. Together, these studies suggest that *T*. *gondii* uses multiple effector proteins to exploit host ESCRTs for vesicular uptake or PV entrapment of host-derived resources.

*T*. *gondii* also interacts with TSG101 and ALIX during parasite invasion into host cells, in this case using RON proteins derived from the necks of rhoptry secretory organelles [98]. The role of TSG101 and ALIX in parasite invasion, however, does not appear to be linked to their function in endosomal sorting since other components of ESCRT-I, ESCRT-III, or VPS4 were absent at the site of parasite invasion [98]. Thus, *T*. *gondii* appears to have multiple strategies for exploiting the host ESCRT machinery at different steps during infection, serving as an interesting model for studying these microbe–host interactions.

### Could there be a role for the ESCRT machinery in immunity?

A significant discovery for the multiple functions of the ESCRT has been its role in repairing membrane damage to protect cancer cells targeted by cytotoxic T cells [149]. The ESCRT machinery can negatively regulate apoptosis, necroptosis, pyroptosis, and ferroptosis to promote cell survival through different mechanisms [150]. For example, disruption of ESCRT function increased inflammasome activation in response to pathogen-associated molecular patterns (PAMPs), leading to pyroptotic cell death [151]. This is due to the anti-inflammatory role of the ESCRT machinery in repairing gasdermin D (GSDMD) pores at the host plasma membrane [151]. Since some of these cell death pathways are activated in infected cells to limit pathogen replication, pathogens could modulate host ESCRTs as a potential immune evasion strategy. Conversely, ESCRT function in membrane repair could be a mechanism for immune cell protection from microbial pore forming proteins that cause membrane lesions. An example of the latter has been reported in the context of *Candida albicans* infection [152]. To protect themselves from lesions caused by the fungal toxin candidalysin, epithelial cells dispose of damaged membrane in an ALG-2/ESCRT-dependent manner [152]. This resembles the contribution of the ESCRT machinery in plants for the protection against pathogens.

Although these examples correspond to the membrane repair function of the ESCRT machinery, its canonical role in vesicular trafficking could also impact the function of signalling pathways [153]. For example, the ESCRT machinery can terminate STING (stimulator of interferon genes) signalling, an important modulator of the type I interferon immune response [154]. Additionally, HRS, STAM2, TSG101, and VPS4 are important for sorting of ubiquitinated T cell receptor into microvesicles for signal termination [155–157]. The ESCRT machinery also negatively regulates antigen cross-presentation in dendritic cells by repairing phagosomal membrane damage, interrupting the export of antigens to the cytosol [158]. A recent report showed that dendritic cells infected with *T*. *gondii* up-regulate the expression of the ESCRT-III component CHMP4B and that CHMP4B is associated with the PV [159]. The same study showed that blocking cholesterol trafficking impaired MHC-I and MHC-II presentation of parasite antigens, reduced PV recruitment of CHMP4B, and suppressed parasite replication [159]. Although the role of the ESCRT machinery in linking these phenotypes hasn’t been elucidated, it would be interesting to know if up-regulation of ESCRT components negatively regulates antigen presentation.

## Concluding remarks

Evidently, microbial exploitation of the host ESCRT machinery is not limited to viral budding or viruses altogether. Whereas viruses appear to benefit mostly from the vesicular budding features of the host ESCRT machinery, bacteria make use of the membrane remodelling function of the host ESCRT machinery during infection [160]. Future studies should focus on how pathogens recruit the host ESCRT machinery to maintain an intact PCV, as for *C*. *burnetii* infection [94], or promoting the formation of MVB-like compartments encapsulating the bacteria [86,87]. Also, how could this be controlled to avoid expulsion from the cells as occurs for UPEC infection [88,161]? What are the factors dictating the different outcomes?

Another interesting discovery to the field is that a eukaryotic pathogen can manipulate the host ESCRT machinery. Protozoan parasites encode a reduced ESCRT machinery that is still functional in EV biogenesis [162,163]. Why does *T*. *gondii*, encoding ESCRT-homologs and capable of producing EVs, prioritize the use of the host ESCRT machinery when the related parasite *Plasmodium falciparum* secretes its ESCRT homologs in to the host cytosol to promote EV formation [164]? Furthermore, *T*. *gondii* can exploit the host ESCRT machinery for invasion and at least 2 different pathways for nutrient acquisition, could it also be making use of the ESCRT machinery for other purposes? To our knowledge, this is the only microorganism capable of exploiting the host ESCRT machinery for multiple cellular functions. Are there other intracellular protozoan parasites capable of interacting with the host ESCRT machinery or do they rely on their own ESCRT machinery?

The apparent role for the ESCRT machinery in immunity opens the question as to whether pathogens modulate the expression of ESCRT components to influence the outcome of infection. The ESCRT machinery can terminate STING signalling; however, it is also necessary for sorting of foreign DNA into EVs to stimulate STING signalling in bystander cells [154,165]. STING signalling promotes inflammasome activation, which results in the assembly of GSDMD pores at the plasma membrane leading to pyroptotic cell death, a process that is tempered by the ESCRT machinery [151]. Thus, much remains to be understood for the function of the ESCRT machinery in innate immunity, the extent to which this is regulated, and how microbes could be exploiting this.

**Fig 6:**
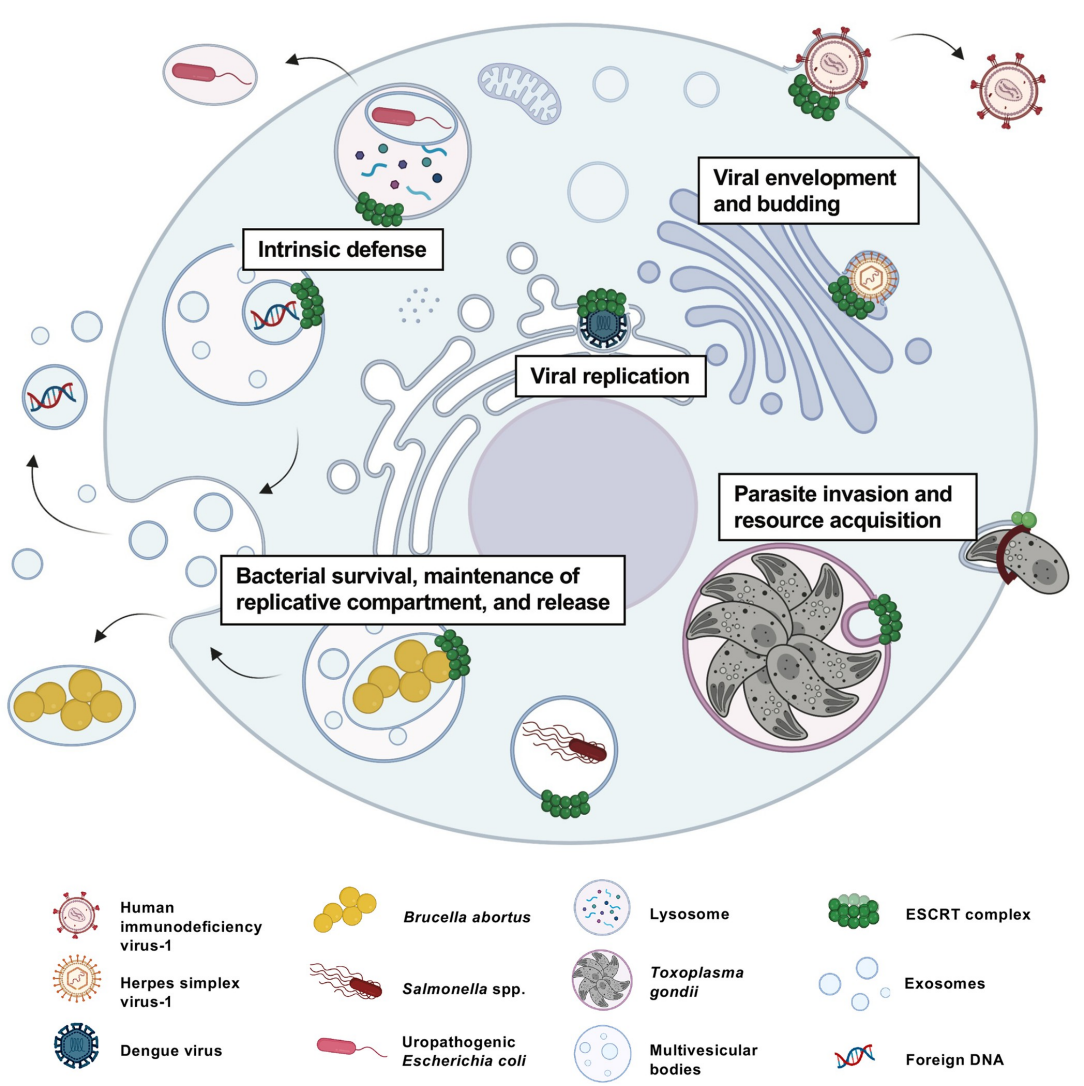
A pathogen’s guide for exploiting the host ESCRT machinery. ESCRT are involved in multiple functions in cells and many intracellular pathogens have evolved ways to exploit ESCRTs for key aspects of their pathogenesis. *Viral envelopment and budding*: For viral envelopment and budding, viruses encode short linear amino acid motifs that mimic ESCRT interactions necessary for ESCRTs sequentially assemble. *Viral replication*: Alternatively, viruses can recruit host ESCRT components for the formation of replication complexes at host organelles. These strategies used by RNA and DNA viruses resemble MVB formation by ESCRT. *Bacterial survival*, *maintenance of replicative compartment*, *and release*: Bacteria can also benefit from the host ESCRT machinery for the biogenesis, maintenance (ESCRT membrane repair function), and release of their replicative compartment (ESCRT MVB formation and exosome release). *Parasite invasion and resource acquisition*: The protozoan parasite *Toxoplasma gondii* recruits early ESCRT components for invasion and subsequently during replication for resource acquisition. *Intrinsic defense*: Interestingly, the role of ESCRT has also been associated with intrinsic defenses against pathogens, for example, expulsion of infecting bacteria and enclosing foreign DNA in exosomes for immune signalling. Created using Biorender.com. ESCRT, Endosomal Sorting Complex Required for Transport; MVB, multivesicular body.

Fundamental knowledge of ESCRT biology has been gained through the study of this host machinery during viral infection. Most notably, identifying the P[S/T]AP late domain motif in HIV-1 Gag and its interaction with TSG101 preceded the realization that these motif were present in HRS and ALIX and that retroviruses were “mimicking” intercomplex interactions to make use of the host ESCRT machinery [13,166]. With the many other pathogen–ESCRT interactions that been identified but not mechanistically understood (**Fig 6**), a treasure trove of insight is likely yet to be gained about how pathogens exploit ESCRTs, thereby exposing new basic mechanisms of this remarkably versatile membrane remodelling machinery.
